# Dental Care Needs and Treatment Priorities in a Homeless Population in Rome: An Observational Study

**DOI:** 10.3390/dj14060330

**Published:** 2026-06-01

**Authors:** Roberta Lione, Francesca Chiara De Razza, Roberto Morello, Massimo Ralli, Giuseppe D’Amato, Giovanni Romano, Manuele Mancini, Paola Cozza

**Affiliations:** 1 Department of Health Sciences, UniCamillus-Saint Camillus International Medical University, 00131 Rome, Italymanuele.mancini@unicamillus.org (M.M.);; 2 Department of Oral and Maxillofacial Sciences, Sapienza University of Rome, 00161 Rome, Italy; 3 Private Practitioner, 00131 Rome, Italy

**Keywords:** dental care, homeless health promotion, oral health, quality of life, prosthetic rehabilitation, removable dentures, dental extractions

## Abstract

**Objectives:** This study aimed to identify the oral health conditions of homeless individuals in Rome, the most frequently required dental treatments, and to describe a standardized, replicable clinical protocol tailored to the specific needs and access barriers of this vulnerable population. **Methods:** Five hundred homeless individuals received comprehensive dental examinations at the Primary Care Services of the Dicastery for the Charity Services (Vatican City) between September 2023 and January 2026. Clinical assessments included oral hygiene status, periodontal health, caries prevalence, and degree of edentulism. Treatment interventions were programmed by scheduling subsequent appointments. For patients requiring prosthetic rehabilitation, treatment was sequenced into distinct steps: preparatory treatments (hygiene, extractions, conservative procedures), impression taking, prosthesis try-in, and delivery. **Results:** Oral health assessment revealed poor or absent hygiene (85.4%), high DMFT scores (63.0%), and root residues (22.4%). Periodontal disease affected 94.0% of participants (gingivitis 73.0%, periodontitis 21.0%). Tooth loss patterns included partial edentulism (12.0%) and complete edentulism (24.0%). A total of 440 appointments were scheduled, with an attendance rate of 78.4%. Prosthetic rehabilitation was completed in 150 patients: 50 received partial dentures (33.3%) and 100 complete dentures (66.7%). **Conclusions:** The examined homeless individuals experienced severe oral health deterioration characterized by extensive tooth loss and advanced periodontal disease. A substantial prosthetic rehabilitation was needed in this sample. The proposed sequential treatment protocol demonstrated high feasibility and patient adherence in this vulnerable population. Comprehensive dental services that address both immediate emergency needs and long-term rehabilitative care are crucial for improving oral health-related quality of life and facilitating social reintegration. Patient-reported outcomes indicated meaningful improvements in digestive function, aesthetic satisfaction, and employment opportunities following prosthetic rehabilitation.

## 1. Introduction

Despite oral health representing a fundamental component of overall health and well-being, vulnerable populations experience oral health burdens and face substantial barriers in accessing preventive and restorative dental care. When compared to the general population, homeless individuals demonstrate higher rates of dental caries, periodontal diseases, and tooth loss [1,2,3,4,5,6,7].

The absence of teeth compromises masticatory function, limiting dietary choices and contributing to malnutrition [8]. Furthermore, the condition of homelessness includes limited access to oral hygiene resources, poor nutrition, and lack of preventive care, thus accelerating oral health deterioration [8,9,10]. This phenomenon can affect speech, social interactions, and employment prospects, creating serious obstacles to consistent integration into society, further marginalizing an already vulnerable population [11,12,13,14,15].

The epidemiology of homelessness in Italy indicates approximately 96,197 individuals throughout the country, with Rome hosting the largest homeless population (22,000 individuals) [16]. However, these numbers likely underestimate the true prevalence due to the heterogeneous living situations, ranging from rough sleeping to temporary shelters, and the transient nature of homelessness [17].

Barriers to dental care for homeless populations are not only economic: beyond financial concerns, homeless individuals encounter additional obstacles including lack of fixed addresses for service registration, difficulty maintaining scheduled appointments due to unstable housing and competing survival priorities that deprioritize oral health [18,19,20].

In response to these difficulties, the Primary Care Services of the Dicastery for the Charity Services, located in the Vatican City, provides comprehensive healthcare services, including dental care, to homeless and marginalized populations [20]. Among the different medical specialties, the Primary Care Services have offered since 2023 free dental visits to the homeless population, to face the limits that typically impede these individuals from accessing basic health services. In the field of dentistry, this structure operates in collaboration with affiliated facilities providing specialized treatments: extractions and oral hygiene at “Madonna Della Fiducia” Clinic, conservative treatments and surgical procedures at Dispensario Santa Marta, and prosthetic rehabilitation at Vincentian Solidarity Dental Centre until December 2024. Since January 2025, prosthetic rehabilitation services have been provided directly by the Primary Care Services, supported by a dedicated dental laboratory. A preliminary epidemiological investigation was conducted in 2024, documenting poor oral health conditions in an observed sample of homeless individuals and the necessity to actively help this vulnerable population maintain a healthier oral status [20].

Several studies focused on the importance of developing dental services addressing homeless populations’ unique needs [21,22,23]. A recent investigation identified the key principles for homeless dental services: patient-centred design, safe and welcoming environments, trained and consistent staff, focus on education, and peer support mechanisms [22]. Successful service models must address both population-level barriers (chaotic lifestyles, anxiety, knowledge gaps) and system-level barriers (inflexible appointments, registration requirements, staff attitudes) [21,22].

However, due to the difficulty of observing the homeless population and the heterogeneous nature of this phenomenon, studies have focused on limited data. Previous investigations about oral health conditions referred to different countries in Europe [21,22,23,24], while a comprehensive characterization of specific oral treatment needs in Italian homeless populations remains limited.

This retrospective observational study aimed to characterize the oral health status and treatment needs of homeless individuals accessing a free dental service in Rome to evaluate the feasibility and outcomes of a standardized sequential treatment protocol, and to assess prosthetic rehabilitation completion rates and patient-reported outcomes in this vulnerable population.

## 2. Materials and Methods

This retrospective observational study was conducted following the Declaration of Helsinki and received approval from the Institutional Ethics Committee of the UniCamillus-Saint Camillus International Medical University (Protocol Number E00486-2026, 6 March 2026). All participants provided written informed consent before enrolment and were informed that their anonymized data would contribute to this research.

The study included adult patients (≥18 years) experiencing homelessness and visiting the Primary Care Services of the Dicastery for the Charity Services, Holy See, for a dental evaluation between September 2023 and January 2026. Participants were enrolled using a consecutive sampling method, including all homeless individuals who accessed the Primary Care Services during the study period and met the eligibility criteria.

Participants were defined as homeless according to the European Typology of Homelessness and Housing Exclusion (ETHOS) classification: Sheltered Homelessness, including individuals residing in homeless shelters for a minimum of 7 consecutive days; Unsheltered Homelessness, referring to people living rough on the streets or in places unsuitable for human habitation for at least 120 non-consecutive days in the preceding 6 months [25]. Exclusion criteria included age < 18 years or failure to meet the ETHOS criteria for homelessness.

Two trained dental examiners (P.C., R.L.) conducted a comprehensive initial dental examination.

A standardized digital medical record collected the general anamnestic information, including demographic data (name, age, sex, country of origin, years residing in Italy), housing status, employment situation, behavioural risk factors (tobacco and alcohol consumption), systemic pathologies, and current medications.

Clinical oral examinations assessed oral hygiene status based on Plaque Index, Bleeding on Probing (BOP), DMFT [20,25,26,27].

Plaque Index evaluated plaque thickness at the gingival margin on a scale from 0 to 3 (0–1: good; 2: poor; 3: absent) [25]. Bleeding on probing (BOP) was recorded according to Ainamo and Bay criteria [26], noting the presence or absence of bleeding within 30 s of probing.

Absent oral hygiene was reported when there was a plaque index of 4 to 5, BOP on more than twenty dental sites, and an incidence of more than six dental caries and/or teeth loss; poor oral hygiene was detected when there was a plaque index of 2 to 3 and BOP on more than ten dental sites with an incidence of four to six dental caries and/or tooth loss; oral hygiene was considered good if the plaque index was 0 or 2 in presence of maximum ten sites of BOP and one to three dental caries or dental loss [20].

Caries and missing teeth were evaluated using the DMFT index according to WHO guidelines [27].

Emergency conditions including acute infections, functional limitations, and pain symptoms received priority assessment and immediate treatment planning.

**Figure 1:**
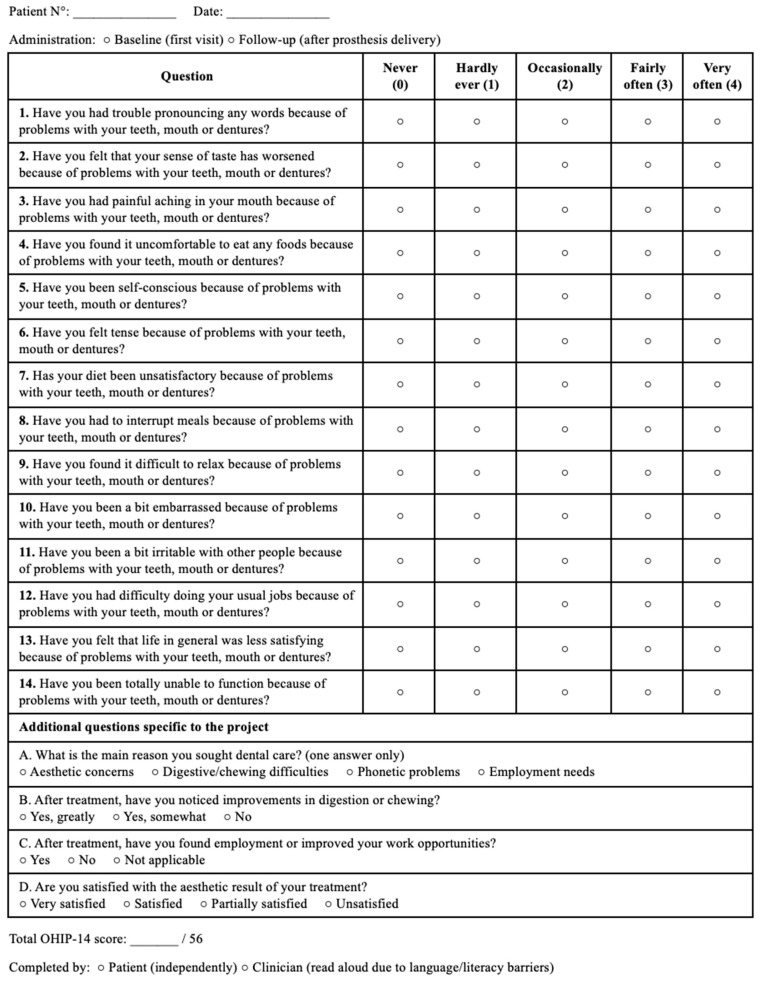
Oral Health Profile questionnaire administered to patients.

During the initial visit, patients were provided with a questionnaire (Oral Health Impact Profile OHIP-14) [28] to assess their primary motivation for seeking dental care, including aesthetic, phonetic, digestive, and employment-related concerns. Given the language barriers and low literacy levels characteristic of this population, the questionnaire was administered verbally by the clinician rather than as a self-administered tool. (Figure 1).

Subsequent appointments were scheduled at appropriate affiliated facilities according to the treatment step required. All treatments were fully covered by the Vatican Service, with help from volunteer healthcare professionals, thus being free for the patients involved in the project. Extractions and oral hygiene were provided at Madonna Della Fiducia Clinic, and conservative treatments and surgical procedures at the Dispensario Santa Marta.

Regarding prosthetic rehabilitation services, they were provided directly by the Primary Care Services. Based on clinical findings and diagnosed dental disorders, individualized treatment plans were developed and sequenced into distinct steps for patients identified as candidates for prosthetic rehabilitation, as represented in Table 1:

Step 1: Hygiene, Dental extractions, conservative treatments, endodontic procedures, and management of acute infections.

Step 2: Impression taking for prosthetic rehabilitation.

Step 3: Prosthetic try-in.

Step 4: Prothesis delivery.

The latter two steps were combined whenever possible.

Following prosthesis delivery, approximately one month later the prothesis delivery, a follow-up questionnaire (OHIP-14, Figure 1) was administered to patients to assess treatment outcomes, including improvements in employment status and digestive function.

### Statistical Analysis

Inter-examiner reliability was assessed on a subset of 30 patients. Both examiners performed independent clinical assessments simultaneously during the first visit, without knowledge of each other’s scores. Inter-examiner agreement was evaluated using Cohen’s Kappa coefficient (κ), calculated for each clinical index after categorization of continuous variables according to the diagnostic thresholds used in the study (DMFT: high ≥ 14/low < 14; BOP: high > 20 sites/moderate 10–20 sites/low < 10 sites; Plaque Index: good 0–1/poor 2/absent 3).

Clinical data were extracted from the internal digital medical records system and exported to Microsoft Excel (Microsoft Corp., Redmond, WA, USA) for database management.

To investigate associations between categorical variables, Pearson’s Chi-Square test (χ^2^) was applied to contingency tables. The analysis examined the relationship between behavioral habits (smoking status, alcohol consumption) and demographic characteristics (age, sex, nationality) with three oral health outcomes: Bleeding on Probing (BOP > 20%), Decayed Missing and Filled Teeth index (DMFT > 14), and Plaque Index. For each test, the Chi-Square statistic, degrees of freedom (df), and *p*-value are reported. A *p*-value < 0.05 was considered statistically significant. All statistical analyses were performed using GraphPad Prism version 9 (GraphPad Software LLC, Boston, MA, USA).

## 3. Results

Cohen’s Kappa coefficients confirmed adequate inter-examiner reliability across all clinical indices (DMFT κ = 0.933, BOP κ = 0.734, Plaque Index κ = 0.609).

Table 2 shows the demographic distribution and the entire description of the sample. The study population comprised 337 males (67.4%) and 163 females (32.6%). The mean age was 51 years (SD = 13 years, range 27–83 years). 330 individuals (66.0%) were non-Italian citizens, while 170 participants (34.0%) were Italian nationals.

Among the 330 non-Italian participants, geographical origins were distributed as follows: 135 individuals (40.9%) from Eastern Europe, 123 individuals (37.3%) from Africa, 45 individuals (13.6%) from Southern Europe (excluding Italy), and 27 individuals (8.2%) from South America.

Regarding behavioural risk factors, substance use was highly prevalent: 197 individuals (39.4%) reported tobacco smoking, 105 individuals (21.0%) consumed alcohol regularly, 155 individuals (31.0%) reported both tobacco and alcohol use, while only 43 individuals (8.6%) reported no substance use.

Systemic health conditions were documented in 66.2% of participants (Table 2). The most frequently reported comorbidities included hypertension (29.6%, *n* = 148), pulmonary diseases (18.0%, *n* = 90), diabetes mellitus (14.0%, *n* = 70), thyroid disorders (7.0%, *n* = 35), psychological/psychiatric conditions (6.4%, *n* = 32), osteoporosis (5.0%, *n* = 25), and HIV infection (2.6%, *n* = 13).

Gastrointestinal disorders were documented in 168 participants (33.6%), with complaints including recurrent gastroenteritis, dyspepsia, and other digestive disturbances. These conditions were frequently attributed by the patients to impaired masticatory function due to extensive tooth loss and inability to properly chew food, resulting in inadequate food breakdown before swallowing.

Clinical examination revealed severe oral health deterioration across the study population (Table 3).

Oral hygiene assessment demonstrated critically poor conditions in most participants. Absent oral hygiene was documented in 247 individuals (49.4%), poor oral hygiene in 180 individuals (36.0%), while only 73 individuals (14.6%) maintained good oral hygiene practices.

The DMFT index revealed a substantial cumulative dental disease burden. High DMFT scores (≥14) were present in 315 participants (63.0%), while 185 participants (37.0%) demonstrated low DMFT scores (<14). Among the decayed teeth component, 65 participants (13.0%) had 1–3 carious teeth, 22 participants (4.4%) had 4–6 carious teeth, and 18 participants (3.6%) presented with more than 6 untreated carious lesions.

The BOP assessment demonstrated widespread gingival inflammation across the cohort. Extensive BOP affecting more than 20 dental sites was documented in 260 patients (52.0%), moderate BOP (10–20 sites) in 162 patients (32.4%), while only 78 patients (15.6%) exhibited minimal BOP (≤10 sites). The high prevalence of extensive BOP correlated with the poor oral hygiene conditions and advanced periodontal disease observed in this vulnerable population.

Root residues with complete crown destruction affected 112 patients (22.4%). Among these, 35 patients (7.0%) had 1–3 root residues, 47 patients (9.4%) had 4–6 root residues, and 30 patients (6.0%) presented with more than 6 root residues.

Acute dental infections and emergency conditions were common. Dental abscesses were diagnosed in 52 patients (10.4%), pulpitis in 27 patients (5.4%), and dental fractures in 25 patients (5.0%).

Regarding periodontal diseases, gingivitis affected 365 patients (73.0%), while more severe periodontitis was diagnosed in 105 patients (21.0%). Advanced periodontal disease with tooth mobility was present in 40 patients (8.0%), indicating terminal periodontal breakdown and imminent tooth loss. Additionally, 22 patients (4.4%) presented with unusual oral lesions requiring further diagnostic investigation and specialist referral.

Among the 500 participants, 40 patients (8.0%) had lost 1–2 teeth, 60 patients (12.0%) presented with partial edentulism affecting multiple teeth or dental arches, and 120 patients (24.0%) had complete edentulism of one or both arches.

Analysis of demographic variables revealed significant associations between age and all three oral health outcomes (Table 4). Younger participants (age < 50 years) showed higher prevalence of BOP > 20% compared to older participants (*p* = 0.047), higher DMFT > 14 (*p* = 0.018), and higher Plaque Index = 3 (*p* = 0.02). No statistically significant associations were found between sex and any of the three outcomes. Similarly, nationality did not significantly influence BOP or DMFT, although a significant difference in Plaque Index distribution was observed between Italian and foreign-born participants (*p* = 0.01), with foreign-born individuals showing a higher proportion of Plaque Index = 3.

Chi-Square analysis of behavioural habits revealed highly significant associations with all three oral health outcomes (Table 5). Regarding smoking status, smokers demonstrated worse periodontal conditions compared to non-smokers, presenting with BOP > 20% (*p* = 0.001). Similarly, DMFT scores were higher in smokers compared to non-smokers (*p* = 0.01). Plaque Index distribution also differed significantly: smokers presented with Plaque Index = 3 (*p* = 0.001).

Alcohol consumption was similarly associated with poorer oral health outcomes: 176 out of 260 alcohol consumers had BOP > 20% versus 85 out of 240 non-drinkers (*p* = 0.02); high DMFT was recorded in 197 out of 258 alcohol consumers compared to 118 out of 242 non-drinkers (*p* = 0.023); and Plaque Index = 3 was present in 170 out of 258 alcohol consumers versus 76 out of 242 non-drinkers (*p* = 0.001).

Treatment needs assessment (Table 6) revealed that only 60 patients (12.0%) required no immediate dental intervention, while 440 patients (88.0%) necessitated one or more treatment modalities.

Pharmacological management for pain and infection control was necessary for 47 patients (9.4%) presenting with acute conditions requiring immediate symptomatic relief before definitive treatment could be initiated. Radiographic examinations were indicated for 22 patients (4.4%) to assess periapical pathology, bone levels, or unusual lesions requiring diagnostic clarification.

Following initial evaluation, a total of 440 appointments were scheduled, with an attendance rate of 78.4% (345 appointments attended). Professional oral hygiene and tartar removal were required by 305 patients (61.0%). Dental extractions were indicated for 335 patients (67.0%), with an average of 4 dental extractions per patient (range 1–14 teeth). Conservative dental treatments (restorations, endodontics) were needed by 45 patients (9.0%). The missed appointment rate was 21.6% (95 appointments).

**Figure 2:**
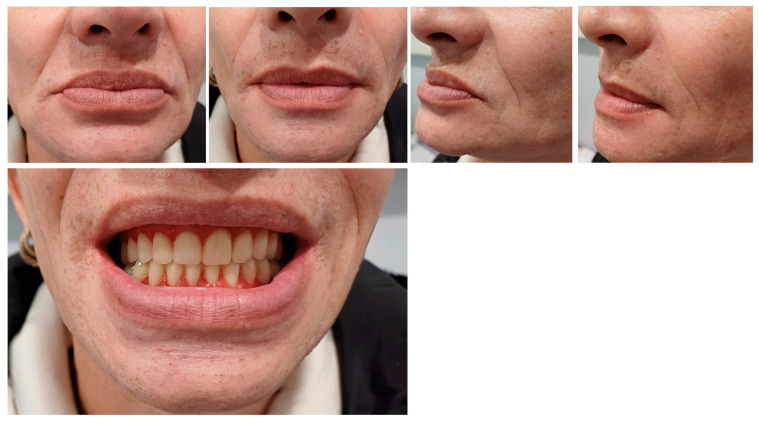
Case Report of Prosthetic Rehabilitation.

**Figure 3:**
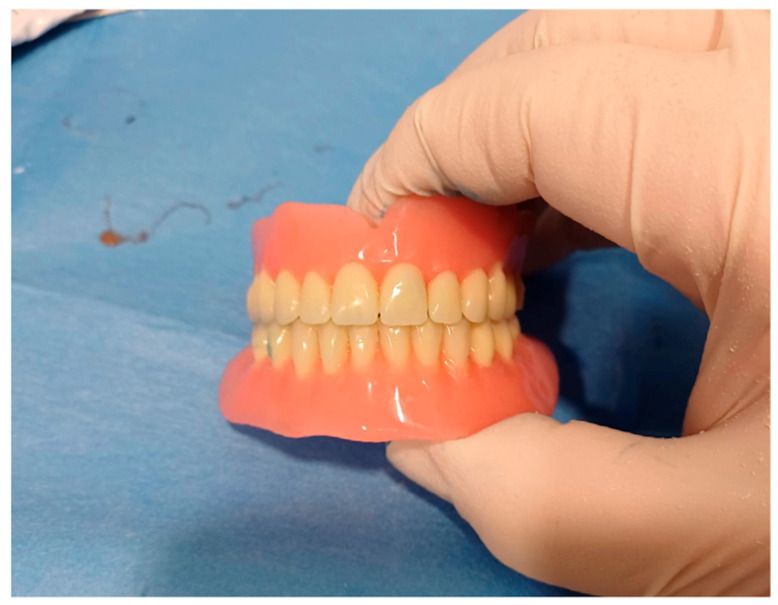
Example of Total Dental Prothesis.

Regarding prosthetic rehabilitation needs, 180 patients (36.0%) were identified as requiring removable dentures for restoration of partially or fully edentulous arches. Of these, 10 patients (5.6%) did not complete treatment due to missed appointments or loss to follow-up during the clinical phases (steps 1–2). A total of 170 prostheses were successfully fabricated through step 3 (impression taking); however, 20 patients (11.1%) did not collect their completed prostheses despite successful fabrication, failing to attend steps 4–5 (try-in and delivery). Finally, 150 prostheses were delivered during the study period. Of the 150 patients who successfully received prosthetic rehabilitation, 60 (40.0%) were female and 90 (60.0%) were male, with a mean age of 45 years (range 38–56 years) Specifically, 50 patients (33.3%) received removable partial dentures (RPDs) for partially edentulous arches, while 100 patients (66.7%) received complete dentures (CDs) for fully edentulous arches (Figure 2 and Figure 3).

Prosthesis loss or theft occurred in 5 patients (3.3% of those who received prostheses), who were offered the opportunity for a one-time prosthesis replacement following the same sequential protocol.

According to the initial questionnaire given during the first visit, the primary motivations for seeking dental care were aesthetic concerns in 195 patients (39%), digestive difficulties in 168 patients (33.6%), employment-related needs in 68 patients (13.6%), and phonetic problems in 42 patients (8.4%) (Table 7).

Among the 150 patients who received prostheses, 118 (78.7%) completed the follow-up questionnaire. Regarding digestive improvements, 82 patients (69.5%) reported better digestive function and reduced gastrointestinal discomfort. Concerning employment outcomes, 23 patients (19.5%) reported having found employment or improved work opportunities following prosthetic rehabilitation. Additionally, 95 patients (80.5%) expressed high satisfaction with aesthetic outcomes, and 71 patients (60.2%) reported improved phonetic function and communication abilities (Table 7).

## 4. Discussion

This observational study aimed to assess the oral health conditions and prosthetic needs of homeless individuals in Rome, and to describe the implementation of a standardized, sequential treatment protocol designed to address the specific barriers this vulnerable population faces in accessing dental care, with particular focus on prosthetic rehabilitation outcomes. The findings demonstrate that homeless individuals experienced a concrete oral health deterioration, marked by poor oral hygiene, advanced periodontal disease, extensive dental caries, and significant tooth loss, thus requiring a significant intervention.

Literature documents severe dental disease among homeless populations in concordance with our findings [29,30,31,32,33,34,35,36,37,38,39,40,41,42,43,44,45,46,47,48].

The analysis of demographic variables revealed significant associations between age and all three oral health outcomes (Table 4). The participants with age under 50 years (225 individuals) showed higher prevalence of BOP, a higher DMFT and worse Plaque Index distribution when compared to the elder individuals (275 people). The homelessness condition among younger individuals contributed to worsen the oral health conditions at an earlier age [30]. No statistically significant associations were found between sex and any of the oral health outcomes, suggesting that oral health deterioration in this population is driven primarily by behavioural and social factors rather than biological sex differences.

Regarding nationality, Italian and foreign-born participants showed comparable BOP and DMFT distributions, while there was a significant difference in Plaque Index. Indeed, foreign-born individuals presented with a worse Plaque Index, suggesting that inadequate access to oral hygiene instruction prior to accessing care may represent a modifiable risk factor in this subgroup, potentially reducing the risk of periodontal disease before it progresses to irreversible stages [31].

The prevalence of poor or absent oral hygiene and high DMFT scores confirms that homeless individuals face substantially greater dental disease burden compared to housed populations. The high prevalence of root residues and acute dental infections suggests that these patients search for dental visit only when experiencing severe pain or functional impairment, by which point dental disease has progressed beyond conservative treatment options [32,33]. Therefore, the high proportion of dental extractions needs (67%) observed in this sample deserves careful interpretation. This finding represents as a direct consequence of delayed presentation and advanced disease burden at the time of first dental contact. The extensive prevalence of root residues (22.4%) and dental abscesses (10.4%), combined with high DMFT scores, indicates that most teeth were beyond conservative salvage when patients first accessed care. This pattern is consistent with the well-documented tendency of homeless individuals to seek dental treatment only when experiencing acute pain or infection, by which point irreversible disease progression has already occurred [34]. Preventive interventions, regular outreach screenings, and integration of dental care within broader social and healthcare services may represent effective strategies to shift the treatment paradigm from predominantly surgical to increasingly conservative approaches in this vulnerable population [34].

The periodontal health status of the observed sample showed a high prevalence of gingivitis and periodontitis. The substantial proportion of patients with tooth mobility indicates advanced periodontal breakdown requiring extraction and prosthetic replacement. The relationship between homelessness, substance abuse, and accelerated periodontal destruction has been well-documented, with homeless individuals demonstrating more rapid disease progression compared to housed individuals with similar risk factors [27,28,29,30]. This investigation confirmed that behavioural habits were strongly associated with periodontal outcomes in the observed sample (Table 4). As expected, smokers and alcohol consumers presented with severe oral conditions (BOP > 20, DMFT > 14, Plaque Index = 3) when compared to people with no behavioural habits.

Therefore, there was a high rate of partial edentulism and complete edentulism. These rates exceed those reported in general adult populations and reflect the cumulative effects of untreated dental diseases, limited preventive care, and prioritization of extractions over conservative treatments when homeless individuals do access dental services [35,36].

Tooth loss profoundly impacts multiple domains of health and quality of life. Functionally, edentulism compromises mastication, limiting dietary choices and potentially contributing to malnutrition—a concern particularly relevant for homeless individuals already facing food insecurity [35]. Nutritional deficiencies can further compromise oral health, systemic health, and wound healing capacity, creating additional treatment challenges [36]. The aesthetic and social impact of tooth loss can be equally devastating, leading to social withdrawal, reduced employment prospects, and psychological distress [37,38].

The findings of this investigation show that 36% of patients required prosthetic rehabilitation. Traditional dental service models for homeless populations have often focused primarily on pain relief and extractions, neglecting the longer-term functional and psychosocial rehabilitation that prosthetic treatment provides [39,40]. These results demonstrate that when prosthetic services are made accessible, uptake is high (83.3% completion rate among identified candidates), suggesting that homeless individuals recognize and value these interventions despite competing life challenges and that appropriately designed services can overcome barriers that typically impede sustained engagement.

Beyond restoring oral function, dentures can significantly improve quality of life, facilitate social interactions, and enhance employment prospects by addressing aesthetic concerns [41,42,43].

This study demonstrates the feasibility and effectiveness of a structured, sequential protocol for prosthetic rehabilitation in homeless populations, addressing both clinical needs and patient-centered outcomes. Of the 180 patients identified as candidates for prosthetic rehabilitation, 150 (83.3%) successfully received their prostheses, representing a high completion rate despite the inherent challenges of treating this vulnerable population.

The treatment attrition occurred primarily at two critical points: 10 patients (5.6%) dropped out during preparatory clinical phases, and 20 patients (11.1%) failed to collect their fabricated prostheses. Despite their motivation to improve their quality of life, some individuals still experience barriers including precarious living conditions and competing survival priorities [44]. The provision of a one-time prosthesis replacement policy was specifically designed to address these contingencies, acknowledging the realities of homelessness while maintaining treatment accessibility [45]. Indeed, the availability of one-time replacement demonstrates the program’s capacity to address the unique challenges faced by homeless individuals, including unstable living conditions and limited storage security.

The severe edentulism and inability to eat properly experienced by many participants was the key for the treatment completion. Moreover, among demographic profile of patients who completed prosthetic rehabilitation revealed a slightly younger cohort (mean age 45 years) compared to the overall study population (mean age 51 years), with a higher proportion of women (40.0% vs. 32.4% in the general sample). in accordance with other studies [46,47,48], oral health needs are fundamental as they might facilitate engagement even in populations typically characterized by poor healthcare utilization.

The patient-reported outcomes stated improvements aligned with their initial motivations: 69.5% experienced better digestive function, 80.5% were satisfied with aesthetic outcomes, and 60.2% noted improved phonetic abilities. Notably, 19.5% of patients reported finding employment or improved work opportunities following rehabilitation, suggesting that dental restoration may contribute to social reintegration by reducing barriers to employment [48]. These findings underscore the importance of accessible prosthetic services as a tool not only for oral health restoration but also for improving overall quality of life and social inclusion in homeless populations.

This observational study has some limitations. The study setting in Rome may limit generalizability to homeless populations in other geographic or healthcare contexts, and individuals entirely disengaged from healthcare services may remain underrepresented. Future studies should consider organic recruitment strategies to provide a more comprehensive picture of oral health needs in the homeless population. Finally, the absence of validated quality of life measures and standardized patient-reported outcome instruments limits our ability to quantify treatment benefits with validated metrics.

## 5. Conclusions

The examined homeless individuals experienced severe oral health deterioration characterized by extensive tooth loss and advanced periodontal disease. A substantial prosthetic rehabilitation was needed in this sample.

The proposed sequential treatment protocol demonstrated high feasibility and patient adherence in this vulnerable population. Comprehensive dental services that address both immediate emergency needs and long-term rehabilitative care are crucial for improving oral health-related quality of life and facilitating social reintegration.

Patient-reported outcomes indicated meaningful improvements in digestive function, aesthetic satisfaction, and employment opportunities following prosthetic rehabilitation.

## Figures and Tables

**Table 1:** Sequential prosthetic rehabilitation protocol implemented for homeless patients.

Step	Procedure	Patients Involved	Goal
1	Oral hygiene instruction and professional scaling, dental extractions, conservative treatments and endodontic procedures, management of acute infections (pharmacological treatment, drainage)	500 patients (all enrolled)	Eliminate active disease, control infection, and achieve oral health stability prior to prosthetic rehabilitation.
2	Impression taking for fabrication of removable partial or complete dentures	180 patients (candidates for prosthetic rehabilitation)	Record accurate impressions and plan the prosthetic design (partial or complete denture) tailored to each patient’s residual dentition and anatomical conditions.
3	Prosthetic try-in	170 patients (prostheses successfully fabricated)	Verify fit, occlusion, aesthetics, and phonetics before final delivery.
4	Prosthesis delivery	150 patients (prostheses delivered)	Deliver the definitive prosthesis, provide oral hygiene and maintenance instructions, and schedule a follow-up appointment approximately one month later for outcome assessment (OHIP-14 questionnaire). Steps 3 and 4 were combined into a single appointment whenever clinically feasible.

Replacement policy: In cases of prosthesis loss or theft (*n* = 5, 3.3%), a one-time replacement was offered following the same sequential protocol, acknowledging the specific challenges of homelessness (residential instability, risk of loss of personal belongings).

**Table 2:** Demographic characteristics, behavioral habits, and systemic comorbidities of the study population (N = 500).

Characteristic	Patients	Percentage
Gender		
Male	337	67.4%
Female	163	32.6%
Nationality		
Italian	170	34.0%
Non-Italian	330	66.0%
Origin (Non-Italian, *n* = 330)		
Eastern Europe	135	40.9%
Africa	123	37.3%
Southern Europe	45	13.6%
South America	27	8.2%
Behavioral Habits		
Tobacco smoking	197	39.4%
Alcohol consumption	105	21.0%
Both tobacco and alcohol	155	31.0%
No substance use	43	8.6%
Systemic Comorbidities		
Hypertension	148	29.6%
Gastrointestinal disorders	168	33.6%
Pulmonary diseases	90	18.0%
Diabetes mellitus	70	14.0%
Thyroid disorders	35	7.0%
Psychological/psychiatric disorders	32	6.4%
Osteoporosis	25	5.0%
HIV infection	13	2.6%
No systemic diseases	169	33.8%

**Table 3:** Oral health status and clinical findings (*n* = 500).

Clinical Parameter	Patients	Percentage
Oral Hygiene Status		
Absent	247	49.4%
Poor	180	36.0%
Good	73	14.6%
DMFT Index		
Low DMFT (<14)	185	37.0%
High DMFT (≥14)	315	63.0%
BOP Index		
High BOP (>20 sites)	260	52.0%
Moderate BOP (10–20 sites)	162	32.4%
Low BOP (<10 sites)	78	15.6%
Decayed Teeth		
1–3 teeth	65	13.0%
4–6 teeth	22	4.4%
>6 teeth	18	3.6%
Missing Teeth		
1–2 missing teeth	40	8.0%
Partial edentulism	60	12.0%
Complete edentulism	120	24.0%
Filled Teeth		
1–3 teeth	2	0.4%
4–6 teeth	15	3%
>6 teeth	10	2%
Root Residues		
1–3 residues	35	7.0%
4–6 residues	47	9.4%
>6 residues	30	6.0%
Total with root residues	112	22.4%
Acute Conditions		
Dental abscesses	52	10.4%
Pulpitis	27	5.4%
Dental fractures	25	5.0%
Periodontal Status		
Gingivitis	365	73.0%
Periodontitis	105	21.0%
Tooth mobility	40	8.0%
Unusual Oral Lesions	22	4.4%

**Table 4:** Association between demographic variables (age, sex, nationality) and oral health outcomes. (Pearson’s Chi-Square test).

Age							
Variables	BOP > 20	BOP ≤ 20		Total	χ^2^	df	*p*-Value
Age < 50 years	136	89		225	10.53	1	0.0047 *
Age ≥ 50 years	125	150		275			
Total	261	239		500			
	DMFT > 14	DMFT ≤ 14		Total	χ^2^	df	*p*-value
Age < 50 years	155	70		225	5.62	1	0.018 *
Age ≥ 50 years	161	114		275			
Total	316	184		500			
	Plaque Index = 1	Plaque Index = 2	Plaque Index = 3	Total	χ^2^	*p*-value	*p*-value
Age < 50 years	22	75	127	224	12.90	0.002 *	0.002 *
Age ≥ 50 years	51	106	119	276			
Total	73	181	246	500			
Sex							
Variables	BOP > 20	BOP ≤ 20		Total	χ^2^	df	*p*-value
Male	184	153		337	2.81	1	0.094
Female	75	88		163			
Total	259	241		500			
	DMFT > 14	DMFT ≤ 14		Total	χ^2^	df	*p*-value
Male	214	123		337	0.21	1	0.646
Female	100	63		163			
Total	313	185		500			
	Plaque Index = 1	Plaque Index = 2	Plaque Index = 3	Total	χ^2^	*p*-value	*p*-value
Male	44	115	178	337	3.53	0.171	0.171
Female	29	64	70	163			
Total	73	179	248	500			
Nationality							
Variables	BOP > 20	BOP ≤ 20		Total	χ^2^	df	*p*-value
Italian	81	88		169	1.24	1	0.265
Foreign-born	178	153		331			
Total	259	241		500			
	DMFT > 14	DMFT ≤ 14		Total	χ^2^	df	*p*-value
Italian	110	60		170	0.59	1	0.443
Foreign-born	203	127		330			
Total	313	187		500			
	Plaque Index = 1	Plaque Index = 2	Plaque Index = 3	Total	χ^2^	df	*p*-value
Italian	35	64	73	172	9.23	2	0.01 **
Foreign-born	40	115	173	328			
Total	75	179	246	500			

χ^2^ = Chi-square value; df = degrees of freedom; BOP = Bleeding on Probing; DMFT = Decayed, Missing, Filled Teeth; * *p* < 0.05; ** *p* < 0.01.

**Table 5:** Association between behavioral habits and oral health outcomes (Pearson’s Chi-Square test).

Smoke Habits							
Variables	BOP > 20	BOP ≤ 20		Total	χ^2^	df	*p*-Value
Smokers	190	12		202	80.18	1	0.001 ***
Non-smokers	62	236		298			
Total	252	248		500			
	DMFT > 14	DMFT < 14		Total	χ^2^	df	*p*-value
Smokers	189	11		200	86.06	1	0.001 ***
Non-Smokers	115	185		300			
Total	304	196		500			
	Plaque Index = 1	Plaque Index = 2	Plaque Index = 3	Total	χ^2^	df	*p*-value
Smokers	2	4	196	202	85.97	2	0.001 ***
Non-smokers	77	171	50	298			
Total	79	175	246	500			
Alcohol habits							
Variables	BOP > 20	BOP ≤ 20		Total	χ^2^	df	*p*-value
Alcohol consumers	176	84		260	49.82	1	0.002 *
Non-drinkers	85	155		240			
Total	261	239		500			
	DMFT > 14	DMFT < 14		Total	χ^2^	df	*p*-value
Alcohol consumers	197	61		258	40.63	1	0.0023 *
Non-drinkers	118	124		242			
Total	315	185		500			
	Plaque Index = 1	Plaque Index = 2	Plaque Index = 3	Total	χ^2^	df	*p*-value
Alcohol consumers	55	33	170	258	97.46	2	0.001 ***
Non-drinkers	24	142	76	242			
Total	79	175	246	500			

χ^2^ = Chi-square value; df = degrees of freedom; BOP = Bleeding on Probing; DMFT = Decayed, Missing, Filled Teeth; Plaque Index. * *p* < 0.05; *** *p* < 0.001.

**Table 6:** Treatment needs and prosthetic rehabilitation outcomes.

Treatment Parameter	Patients	Percentage
No treatment required	60	12.0%
Treatment required	440	88.0%
Specific Interventions Required		
Professional oral hygiene/scaling	305	61.0%
Dental extractions	335	67.0%
Conservative treatments	45	9.0%
Pharmacological management	47	9.4%
Radiographic examination	22	4.4%
Prosthetic Rehabilitation	180	36.0%
Prostheses delivered	15,096	83.3%
Removable Partial Dentures (RPDs)	50	33.3%
Complete Dentures (CDs)	100	66.7%
Lost to follow-up/did not collect	30	16.7%

**Table 7:** Patient-reported motivations and outcomes of prosthetic rehabilitation.

Initial Motivation Questionnaire (*n* = 500)	Patients	Percentage
Aesthetic concerns	195	39%
Digestive difficulties	168	33.6%
Employment-related needs	68	13.6%
Phonetic problems	42	8.4%
Follow-up Questionnaire (N = 118)	Patients	Percentage
Improved digestive function	82	69.5%
Aesthetic satisfaction	95	80.5%
Improved phonetic function	71	60.2%
Found employment/improved opportunities	23	19.5%

## Data Availability

Data are available on request.

## Abbreviations

The following abbreviations are used in this manuscript:

DMFT index	Index of Decayed, Missed, Filled Teeth
BOP index	Bleeding on Probing Index
